# Reprogramming of DNA methylation is linked to successful human preimplantation development

**DOI:** 10.1007/s00418-021-02008-6

**Published:** 2021-06-27

**Authors:** Julia Arand, Renee A. Reijo Pera, Mark Wossidlo

**Affiliations:** 1 Department of Cell and Developmental Biology, Center of Anatomy and Cell Biology, Medical University of Vienna, 1090 Vienna, Austria; 2 Department of Genetics, Stanford University, Stanford, CA 94305 USA; 3 Department of Pediatrics, Stanford University, Stanford, CA 94305 USA; 4 Department of Obstetrics and Gynecology, Stanford University, Stanford, CA 94305 USA; 5 Institute for Stem Cell Biology and Regenerative Medicine, Stanford University, Stanford, CA 94305 USA; 6 Present Address: McLaughlin Research Institute, Great Falls, MT 59405 USA

**Keywords:** DNA methylation reprogramming, Active DNA demethylation, Human preimplantation development, Successful human embryogenesis

## Abstract

Human preimplantation development is characterized by low developmental rates that are poorly understood. Early mammalian embryogenesis is characterized by a major phase of epigenetic reprogramming, which involves global DNA methylation changes and activity of TET enzymes; the importance of DNA methylation reprogramming for successful human preimplantation development has not been investigated. Here, we analyzed early human embryos for dynamic changes in 5-methylcytosine and its oxidized derivatives generated by TET enzymes. We observed that 5-methylcytosine and 5-hydroxymethylcytosine show similar, albeit less pronounced, asymmetry between the parental pronuclei of human zygotes relative to mouse zygotes. Notably, we detected low levels of 5-formylcytosine and 5-carboxylcytosine, with no apparent difference in maternal or paternal pronuclei of human zygotes. Analysis of later human preimplantation stages revealed a mosaic pattern of DNA 5C modifications similar to those of the mouse and other mammals. Strikingly, using noninvasive time-lapse imaging and well-defined cell cycle parameters, we analyzed normally and abnormally developing human four-cell embryos for global reprogramming of DNA methylation and detected lower 5-methylcytosine and 5-hydroxymethylcytosine levels in normal embryos compared to abnormal embryos. In conclusion, our results suggest that DNA methylation reprogramming is conserved in humans, with human-specific dynamics and extent. Furthermore, abnormalities in the four-cell-specific DNA methylome in early human embryogenesis are associated with abnormal development, highlighting an essential role of epigenetic reprogramming for successful human embryogenesis. Further research should identify the underlying genomic regions and cause of abnormal DNA methylation reprogramming in early human embryos.

**Supplementary Information:**

The online version contains supplementary material available at 10.1007/s00418-021-02008-6.

## Introduction

Human preimplantation development is characterized by fecundity rates that are low, largely due to loss of pre- and postimplantation embryos (Macklon et al. 2002; Niakan et al. 2012). In vitro, only 30–50% of human zygotes develop to the blastocyst stage (Gardner et al. 2000). The underlying reason for this developmental incompetence is poorly understood. Previous studies point towards a high rate of aneuploidies (50–80% of human cleavage-stage embryos) as one possible cause (Chavez et al. 2012; Vera-Rodriguez et al. 2015), but the impact of successful epigenetic reprogramming on the developmental competence of human preimplantation embryos is just beginning to be understood.

In early development, major epigenetic reprogramming occurs in humans, as in most mammalian species, although with different dynamics and extent (Messerschmidt et al. 2014; Morgan et al. 2005; Reik et al. 2001). In addition to global replacement of protamines in the male chromatin by oocyte-provided histones and reprogramming of histone modifications specific to the parental genomes (Xia et al. 2019), a major wave of global DNA demethylation is also observed. This global DNA methylation reprogramming begins directly after fertilization and continues through the blastocyst stage, with pronounced first demethylation of the paternal genome in the zygote of many mammals, especially in the mouse, where most studies on DNA methylation reprogramming have been conducted (Dean et al. 2001; Fulka et al. 2004; Mayer et al. 2000; Oswald et al. 2000; Petrussa et al. 2016; Wossidlo et al. 2010). Mechanisms of global DNA methylation reprogramming involve a complex interplay of mainly passive DNA demethylation along with de novo methylation and active DNA demethylation involving oxidation of 5-methylcytosine (5mC) by ten-eleven translocation (TET) enzymes (Amouroux et al. 2016; Arand et al. 2015; Gu et al. 2011; Ladstatter and Tachibana 2019; Wossidlo et al. 2011). It is thought that epigenetic reprogramming of the parental epigenomes ensures the development of totipotent and pluripotent preimplantation embryos (Eckersley-Maslin et al. 2018; Messerschmidt et al. 2014), but the impact of DNA methylation reprogramming on early human embryogenesis is still unclear.

In this study, we analyzed the remodeling of DNA methylation (5mC) in early human preimplantation development to gain further insights into epigenetic reprogramming of human embryos. We characterized the dynamic appearance of 5mC and TET enzyme-mediated DNA modifications [5-hydroxymethylcytosine (5hmC), 5-formylcytosine (5fC), and 5-carboxylcytosine (5caC)] in early human embryos. Furthermore, we hypothesized that reprogramming of DNA methylation is linked to normal human preimplantation development. Thus, we compared normally and abnormally developing human four-cell embryos using noninvasive time-lapse imaging and analysis of global levels of DNA 5C modifications in order to shed light on the role of DNA methylation reprogramming in successful human preimplantation development.

## Materials and methods

### Source of human embryos and ethical approval

All experimental work was performed at Stanford University. Supernumerary human embryos from successful in vitro fertilization (IVF) cycles, donated for non-stem research, were obtained with informed consent from the Stanford University RENEW Biobank. De-identification of samples was performed according to the Stanford University Institutional Review Board approved protocol #10466 entitled “The RENEW Biobank.” No protected health information was associated with individual embryos.

### Human embryo retrieval and culture

Frozen human embryos at different stages of preimplantation development from zygotic up to blastocyst stage were thawed as described previously (Wong et al. 2010). Briefly, cryo-containers were removed from liquid nitrogen and exposed to air for 10 s before incubating in a water bath at 37 °C until thawed. Next, embryos were transferred to 0.5 M and 0.2 M sucrose solution for 10 min each and washed in diluent solution for 10 min at room temperature (RT). The embryos were then incubated in Quinn’s Advantage cleavage medium (CooperSurgical, Trumbull, CT, USA) supplemented with 10% serum protein substitute (CooperSurgical, Trumbull, CT, USA) under mineral oil (Sigma-Aldrich, St. Louis, MO, USA) at 37 °C with 6% CO_2_, 5% O_2_, and 89% N_2_, standard human embryo culture conditions in accordance with current clinical IVF practice.

### Time-lapse imaging of human embryos

Preimplantation development was monitored using a custom-built miniature microscope system that was modified for dark-field illumination as described previously (Wong et al. 2010). Embryos were cultured in custom dishes with individual micro-wells to track single embryos during time-lapse imaging, where all of the micro-wells shared a common media drop under mineral oil to maintain group culture. Images were taken every 5 min up to the early four-cell stage. After each experiment, images were compiled into a time-lapse movie by ImageJ software, and cell cycle parameters were calculated as described previously (Wong et al. 2010).

### Embryo staining and immunofluorescence microscopy

For immunofluorescence (IF) analysis, embryos were briefly washed in Quinn’s Advantage medium with HEPES (CooperSurgical, Trumbull, CT, USA) plus 5% serum protein substitute (CooperSurgical, Trumbull, CT, USA), and zona pellucida was removed by treatment with acidic Tyrode’s solution (Millipore, Darmstadt, Germany). Embryos were fixed for 20 min in 3.7% paraformaldehyde in phosphate-buffered saline (PBS) at 4 °C and permeabilized with 0.2% Triton X-100 (Sigma-Aldrich, Darmstadt, Germany) in PBS for 10 min at RT. Next, embryos were incubated in 4 N HCl solution at RT for 15 min. Following neutralization (10 min, 100 mM Tris–HCl, pH 8.0) and second fixation, embryos were stained with anti-5mC [mouse monoclonal (1:500); Eurogentec, Fremont, CA, USA], anti-5hmC [rabbit polyclonal (1:500); Active Motif, Carlsbad, CA, USA], anti-5fC [rabbit polyclonal (1:500); gift from Yi Zhang (Howard Hughes Medical Institute, Boston Children's Hospital, Boston, MA, USA), validated in Inoue et al. 2011], or anti-5caC [rabbit polyclonal (1:500); gift from Yi Zhang, validated in Inoue et al. 2011]. In addition, selected blastocysts were co-stained with either anti-Nanog [rabbit polyclonal (1:100); ReproCELL, Beltsville, MD, USA] or anti-Oct4 [goat polyclonal (1:200), Santa Cruz Biotechnology, Dallas, TX, USA] to confirm inner cell mass (ICM) localization. Following several washes in blocking solution, embryos were incubated at RT with anti-mouse Alexa Fluor 488 (10 μg/ml; Molecular Probes, Thermo Fisher Scientific, Waltham, MA, USA), anti-rabbit Alexa Fluor 647 (10 μg/ml; Molecular Probes), and anti-goat Alexa Fluor 350 (10 µg/ml; Molecular Probes) secondary antibodies for 2 h. After incubation in propidium iodide (PI) [10 min (2 μg/ml); Sigma-Aldrich, Darmstadt, Germany], embryos were washed and mounted on slides with a small drop of Vectashield (Vector Laboratories, Burlingame, CA, USA) mounting medium. Embryos were analyzed on a Zeiss LSM 510 Meta inverted laser scanning confocal microscope as described previously (Wossidlo et al. 2010). ImageJ software was used to quantify antibody signals of z-stack computed (sum slices, ~ 20 stacks with 0.4 μm per sample) IF images, and antibody signals for DNA modifications were normalized to DNA signal (PI) to account for potential aneuploidies, frequently observed in human preimplantation embryos (Chavez et al. 2012; Vera-Rodriguez et al. 2015).

### Statistical analysis

Statistical analyses were performed using GraphPad Prism 8.0 (GraphPad Software Inc., San Diego, CA, USA).

## Results

### DNA methylation reprogramming in the human zygote

Based on previous studies of mouse, rabbit, and bovine embryos (Dean et al. 2001; Inoue and Zhang 2011; Wossidlo et al. 2011), we addressed whether DNA methylation reprogramming dynamics might be conserved in human preimplantation embryos. We examined 5mC levels simultaneously with 5hmC, 5fC, or 5caC levels by indirect IF with well-characterized antibodies in freshly thawed human zygotes at the G2 phase. The use of IF rather than genomic sequencing methods enables analysis of multiple DNA 5C modifications simultaneously.

**Fig. 1:**
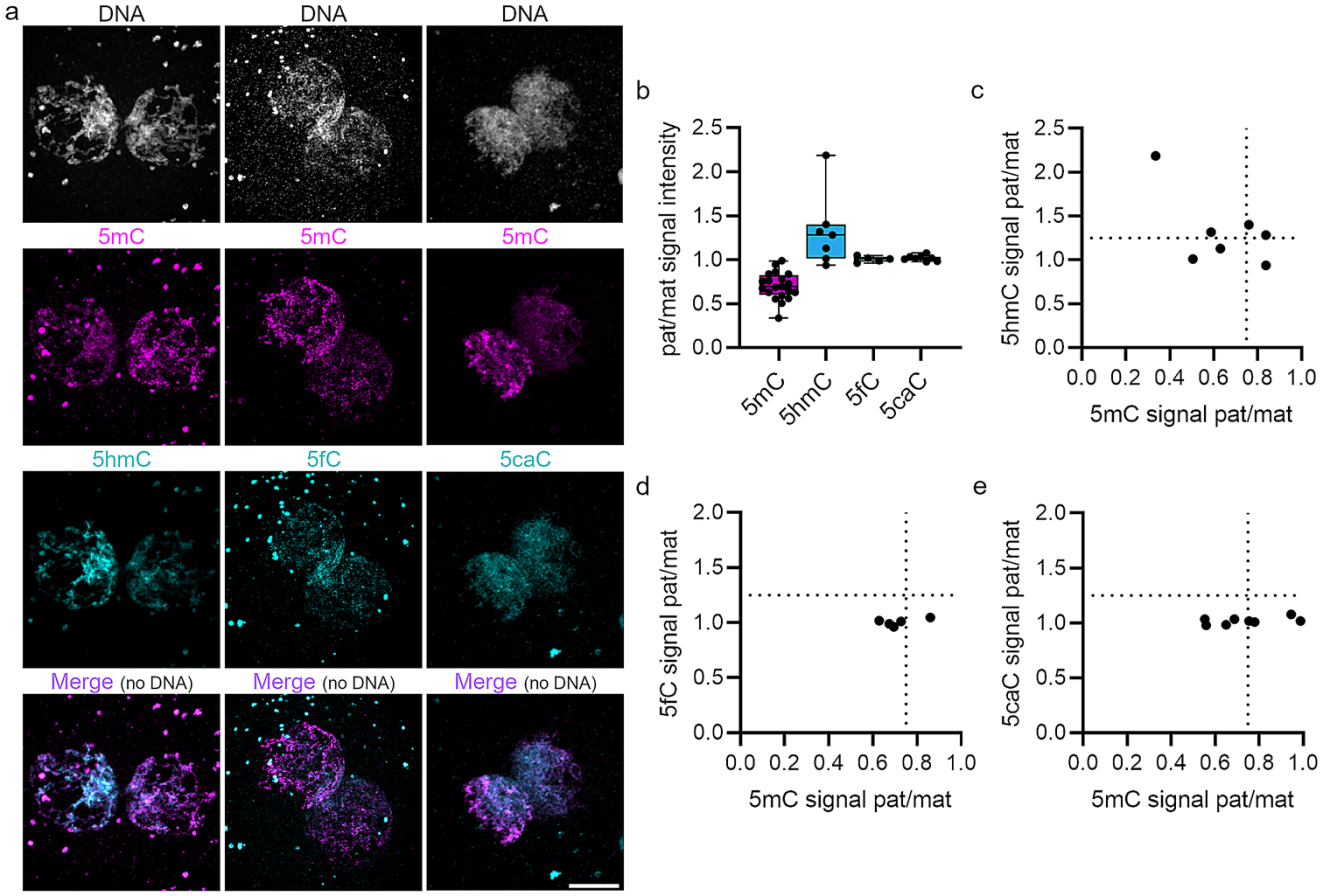
Human-specific dynamics of DNA methylation reprogramming in zygotes. **a** Representative images of indirect immunostaining for 5mC and 5hmC, 5fC, or 5caC in human zygotes at late G2 stage (a total of 5–20 zygotes per DNA modification were analyzed. Scale bar = 20 μm). **b** Quantification of integral IF signal intensities of 5mC (magenta) and derivatives (cyan) in human zygotes at late G2 stage normalized against DNA signal. Shown are paternal versus maternal signal ratios for 5mC, 5hmC, 5fC, and 5caC. Note the high variability in paternal/maternal 5mC and 5hmC signal ratios (each dot in the box plot represents a single embryo). **c**–**e** Scatter plots of 5mC versus 5hmC (**c**), 5fC (**d**), and 5caC signals (**e**). The dotted line on the *x*-axis at 0.75 marks a cutoff for loss of 5mC and the dotted line on the *y*-axis at 1.25 a cutoff for gain of 5hmC/5fC/5caC in the paternal pronucleus

Our analysis indicated several key findings. We observed that all four DNA modifications were present in both parental pronuclei (Fig. 1a) and quantified the ratio of 5C modifications in maternal and paternal pronuclei (Fig. 1b, Supplementary Table S1). Since in human zygotes, in contrast to the mouse, the two parental pronuclei are of similar size, assignment to the parental origins of the pronuclei was determined by their respective levels of 5mC signal. Therefore, the pronucleus with a stronger 5mC antibody signal was defined as the maternal pronucleus (ratio of mat/pat 5mC signal intensity > 1; see also Fulka et al. 2004). Interestingly, loss of 5mC signal in human zygotes is not as strongly pronounced as in mouse zygotes (Fig. 1b). In human zygotes, the paternal versus maternal 5mC signal ratio is on average 0.7, whereas in mouse zygotes the paternal pronucleus becomes more demethylated, with a paternal/maternal 5mC signal ratio of ~ 0.4 (Supplementary Fig. 1; Wossidlo et al. 2011). This observed human ratio is similar to previously published data in human zygotes (Fulka et al. 2004; Petrussa et al. 2016). Moreover, 60% (12/20) of analyzed zygotes showed stronger demethylation in one pronucleus (an asymmetric 5mC staining in the two pronuclei, defined by a paternal to maternal ratio < 0.75) and 40% more similar levels of 5mC (as defined by a ratio > 0.75) in both pronuclei (Supplementary Table 1 and see also Fulka et al. 2004). A small fraction of zygotes (35%), where DNA demethylation of the paternal genome could not be observed, was also reported in bovine embryos, while in contrast mouse zygotes show reproducible demethylation (Hou et al. 2007; Mayer et al. 2000; Santos et al. 2002; Wossidlo et al. 2010, 2011). Next, we analyzed the appearance of 5hmC in human zygotes and found less intense 5hmC signals in the human paternal genome when compared to mouse paternal pronuclei at the late G2 stage (Fig. 1a). We observed on average a paternal/maternal 5hmC signal ratio of 1.2 in human zygotes (Fig. 1b) compared to a ratio of 2.3 in mouse zygotes (Supplementary Fig. 1; Wossidlo et al. 2011). Four out of the seven analyzed human zygotes showed a stronger 5hmC signal in the paternal pronucleus (pat/mat 5hmC ratio > 1.25), whereas the others showed similar levels compared to the maternal pronucleus (ratio of 0.94–1.1, Supplementary Table S1). Notably, both 5mC and 5hmC signals were characterized by a high variance in the detected signal intensities—with paternal/maternal ratios ranging from 0.34 to 0.99 for 5mC and 0.94 to 2.2 for 5hmC (Fig. 1b). Moreover, a weaker 5mC signal in the paternal pronucleus (ratio < 0.75) did not always correlate with a stronger 5hmC signal (ratio > 1.25) in the same zygote (two out of four, Fig. 1c). 5fC showed an overall weak signal in human zygotes, with no apparent difference between the parental pronuclei (pat/mat 5fC signal ratio = 1.01, Fig. 1b, compare also to Gao et al. 2020). 5caC, the last modification step of 5mC generated by TET enzymes, was also detected in both pronuclei of human zygotes, with no difference in paternal versus maternal 5caC signal intensity (pat/mat 5caC signal ratio = 1.02, Fig. 1b). This is in contrast to the mouse, where the paternal pronucleus is strongly stained for 5fC (×2.6) and 5caC (×10) (Supplementary Fig. 1, and Inoue et al. 2011). 5fC and 5caC paternal versus maternal ratios showed a low variance in all analyzed human zygotes and were detected in both 5mC asymmetrically and 5mC symmetrically stained embryos (Fig. 1d, e). In summary, we observed that human zygotes follow human-specific dynamics of DNA demethylation, with variable 5mC and 5hmC appearance, but a similar tendency as in mouse zygotes, with less pronounced 5mC and 5hmC ratios in the parental pronuclei, and in contrast to the mouse, no accumulation of 5fC or 5caC in paternal pronuclei.

### DNA 5C modifications in human cleavage-stage human embryos

**Fig. 2:**
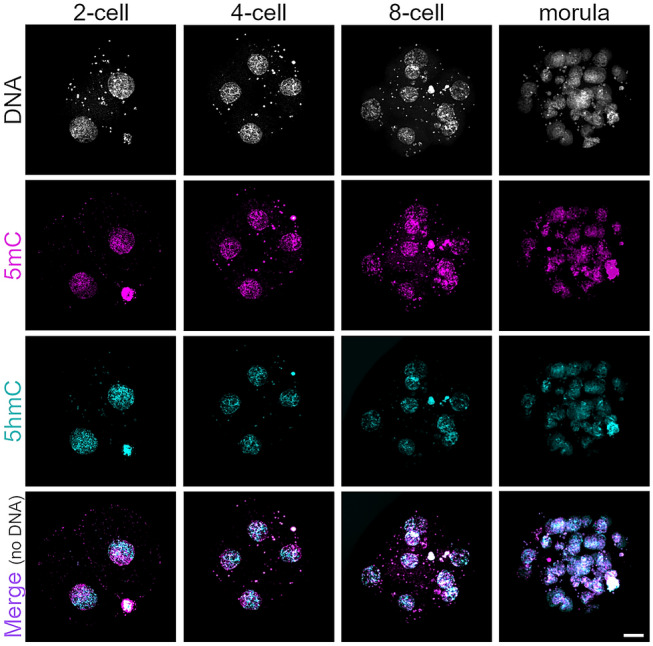
Immunofluorescence analysis of 5mC and 5hmC in human cleavage-stage embryos. Representative immunofluorescence images of human embryos at distinct stages of preimplantation development analyzed for 5mC (magenta) and 5hmC signals (cyan) (*n* = 9, 24, 9, 11 for two-cell, four-cell, eight-cell, and morula-stage embryos, respectively). Scale bar = 20 μm

We next followed 5mC and 5hmC patterns by IF during human preimplantation development and found a comparable dilution of both signals during consecutive cleavage stages up to the morula stage similar to early mouse development (Fig. 2; for mouse see references Inoue and Zhang 2011; Okamoto et al. 2016; Ruzov et al. 2011). In human two-cell embryos, a clear asymmetrical distribution of strong 5mC and 5hmC signals are detectable in eight out of the nine analyzed two-cell embryos, likely representing still separated maternal- and paternal-derived chromatin, as reported for mouse two-cell embryos (Gu et al. 2011). Similar to mouse four-cell embryos and morulas, we also observed a mosaic pattern of IF signals for 5mC and 5hmC in human four-cell and morula-stage embryos, which suggested potential passive dilution of DNA methylation in human preimplantation development similar to the mouse.

**Fig. 3:**
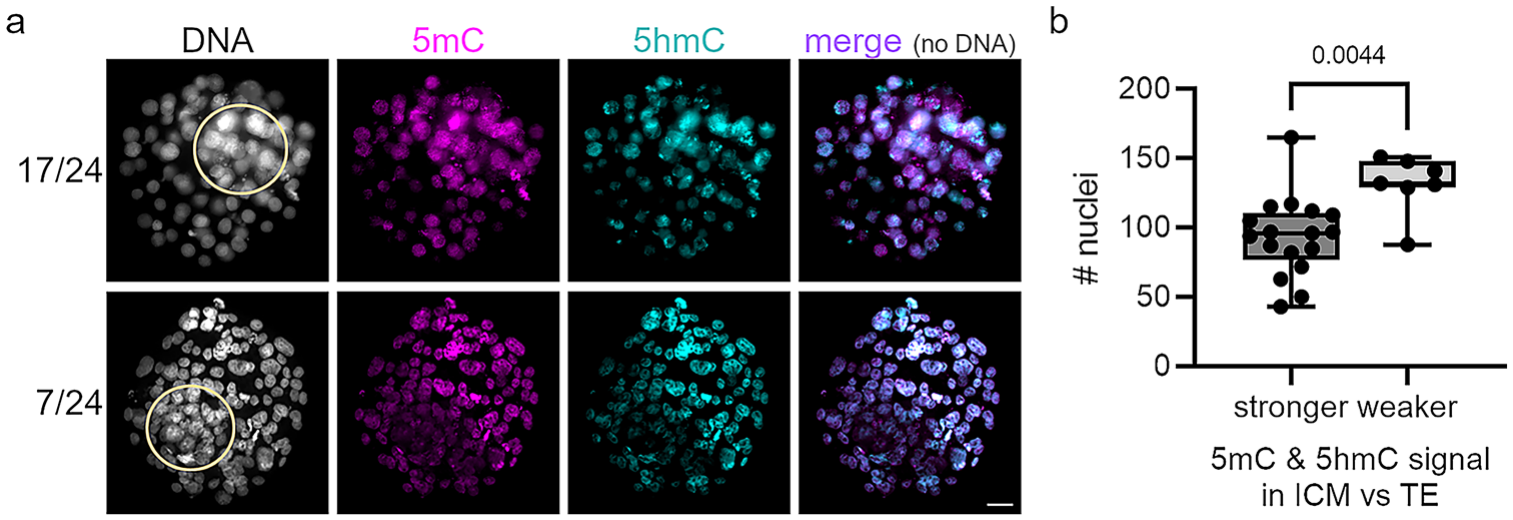
Immunofluorescence analysis of human blastocysts. **a** Representative immunofluorescence images of human blastocysts analyzed for 5mC (magenta), 5hmC(cyan), and DNA signals (white). The major fraction of human blastocysts (17 of 24, upper panel) show stronger 5mC and 5hmC signals in the inner cell mass (ICM) compared to the trophectoderm; a smaller subset (7 of 24, lower panel) show weaker 5mC and 5hmC signals in the ICM compared to trophectoderm. The ICM is indicated by a yellow circle. Scale bar = 20 μm. **b** Number of cells per blastocyst with stronger or weaker 5mC and 5hmC signals in the ICM compared to trophectoderm. Each dot represents a single blastocyst. Statistically significant differences were calculated using two-tailed Student’s *t* test

As preimplantation development is ending, the first lineage commitments begin. The outer cell layer commits to trophectoderm (TE) development, and the small cluster of cells in the inner cavity, the inner cell mass (ICM), commits to ultimately give rise to the embryo proper. At this stage, the lowest amount of DNA methylation can be observed, shortly before tissue-specific DNA methylation patterns of postimplantation development are established (Guo et al. 2014a, b; Smith et al. 2014). We analyzed 24 human blastocytes for 5mC and 5hmC abundance (Fig. 3a). The majority (71%) showed a stronger signal of 5mC and 5hmC in the inner cell mass relative to the TE layer. ICM was defined as the region with a higher abundance of cell nuclei, which was confirmed for selected blastocysts by co-staining with OCT4 or Nanog (Supplementary Fig. 2). A smaller fraction (29%) of the analyzed blastocysts showed lower signals of 5mC and 5hmC in the ICM relative to TE cells. Counting of nuclei in analyzed blastocysts revealed that blastocysts with higher 5mC and 5hmC signals in the ICM had significantly fewer cells than the blastocysts with lower signals of 5mC and 5hmC (Fig. 3b). Hence, these two types represent early- and late-stage developing blastocysts, indicating that in late preimplantation development, global DNA demethylation is still occurring in the pluripotent ICM. The low signal of 5mC/5hmC is in strong contrast to 5mC/5hmC abundance reported in mouse blastocysts, where the ICM shows stronger signals for both compared to TE (Ruzov et al. 2011; Santos et al. 2002). Previous studies also report low 5mC signals in the ICM in humans and other mammals such as bovine (Fulka et al. 2004; Hou et al. 2007).

### 5mC and 5hmC abundance correlates with human early cell cycle parameters

**Fig. 4:**
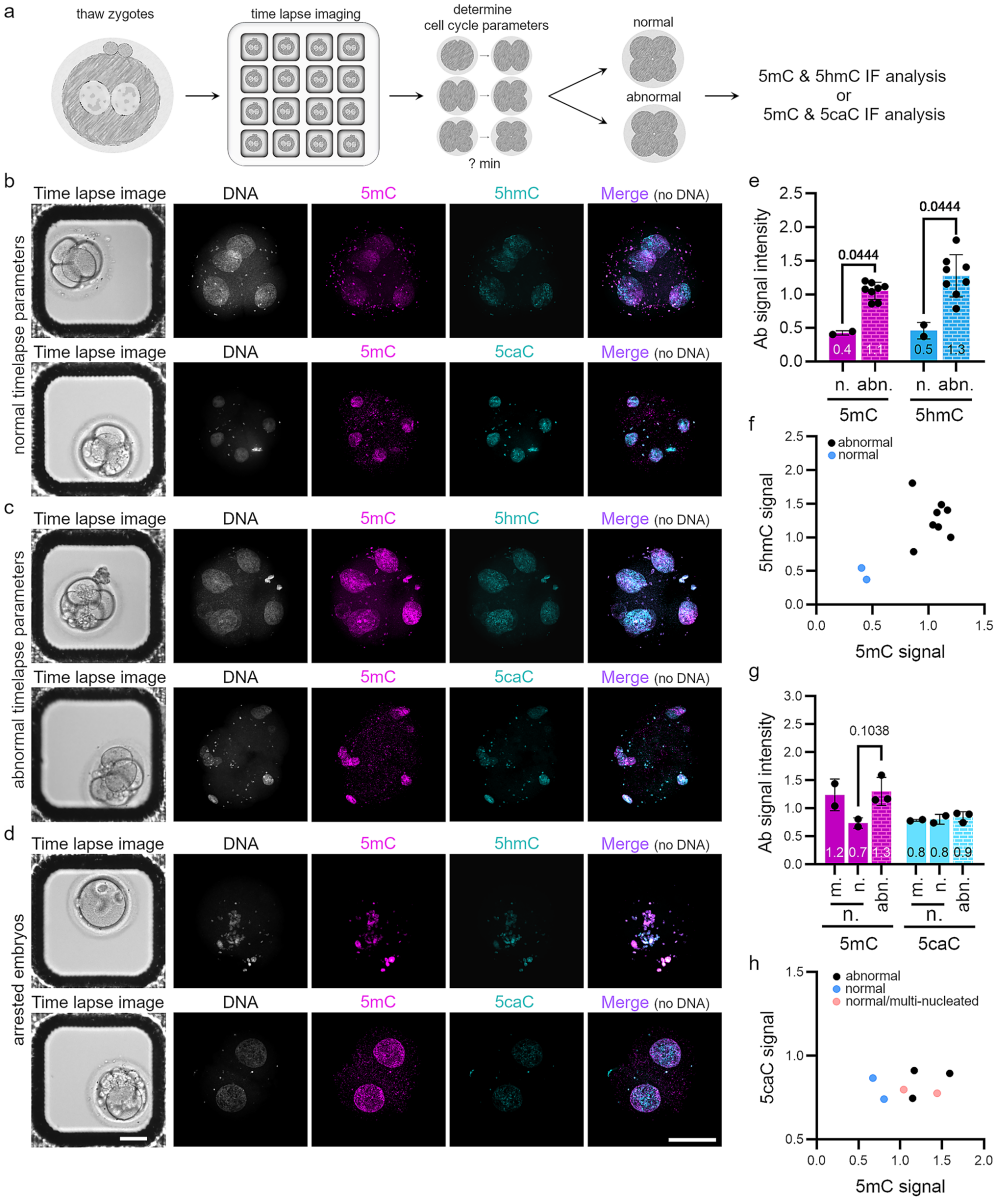
DNA methylation reprogramming in normally and abnormally developing human four-cell embryos. **a** Experimental setup. **b**–**d** Representative immunostaining of human embryos cultured to the four-cell stage by noninvasive time-lapse imaging and analyzed by 5mC, 5hmC, and 5caC IF. **b** Human four-cell embryos predicted to develop to the blastocyst stage via noninvasive time-lapse parameters (see Wong et al. 2010). **c** Human embryos cultured to the four-cell stage and predicted to be nonviable via noninvasive time-lapse parameters and **d** arrested human embryos. Scale bar = 20 μm. **e**–**h** Quantification of 5mC (**e**, **g**), 5hmC (**e**), and 5caC (**f**) signal intensities in normally (n.) and abnormally (abn.) developing four-cell embryos. Antibody signals were normalized to DNA signal. Each dot represents a single embryo. Statistical significance was calculated using the Mann–Whitney test (**e**) or Kruskal–Wallis test (**g**). Correlation of 5mC to 5hmC (**f**) and 5mC to 5caC (**h**) signal intensity in analyzed four-cell embryos. Each dot represents a single embryo; normally developing embryos are shown in blue. m. = multi-nucleated

Given the high degree of variability in global DNA demethylation (5mC and 5hmC levels) in human zygotes and the low developmental success rates of early human embryos, we next investigated the correlation between DNA methylation reprogramming and predicted outcomes of human preimplantation development. For this purpose, we used noninvasive time-lapse imaging of cultured human zygotes to identify normally and abnormally developing human embryos at the four-cell stage (Supplementary Movies S1 and S2). To identify normally and abnormally developing embryos at this early stage, we analyzed three well-defined cell cycle parameters which can predict normal and abnormal human preimplantation development to the blastocyst stage with accuracy of ~ 93% (Wong et al. 2010). We then analyzed 5mC and 5hmC or 5mC and 5caC levels by IF in four-cell embryos and arrested embryos (Fig. 4a–d). All three DNA modifications were detected in normally and abnormally developing four-cell embryos, as well as in embryos arrested earlier (Fig. 4b–d, Supplementary Tables S2 and S3). Similar to the four-cell embryos in Fig. 2, we observed a mosaic pattern for the IF signal of all three analyzed DNA modifications (Fig. 4b, c). Expectedly, abnormally developing embryos revealed a high frequency of blastomeres with more than one nucleus (9/20), consistent with previous observations of a high rate of aneuploid human preimplantation embryos (Chavez et al. 2012; Vanneste et al. 2009; Vera-Rodriguez et al. 2015). Next, we quantified 5mC, 5hmC, and 5caC signal intensities in normally and abnormally developing four-cell embryos. In the first time-lapse experiment, we co-analyzed 5mC and 5hmC levels. The number of normally developing embryos in this group was low (2/13) (Supplementary movie S1, Supplementary Table S2); however, we observed a distinct positive correlation of low levels of both 5mC and 5hmC in normally developing embryos, whereas abnormally developing embryos were characterized by higher 5mC and 5hmC levels (> twofold higher than in normally developing four-cell embryos). In the second time-lapse experiment, we co-analyzed 5mC and 5caC levels by IF. We detected equal staining patterns for 5caC in both parental pronuclei in zygotes (Fig. 1a, b), suggesting only a minor role of 5caC at the zygotic stage in human embryogenesis. We then questioned whether 5caC signals appear stronger in later embryos and whether normally and abnormally developing human embryos differ in 5caC levels at a later stage in preimplantation development. Results indicated that 4/9 embryos developed with normal cell cycle parameters to the four-cell stage (Supplementary movie S2, Supplementary Table S3). Here, we detected very similar levels of 5caC in normal and abnormal embryos (Fig. 4g). In general, the observed 5caC signal was heterogeneously distributed in all nuclei, as previously shown in metaphase spreads of mouse four-cell embryos (Inoue et al. 2011). In this second experiment, we did not observe a significant difference in 5mC levels of normal versus abnormal embryos; however, a similar tendency as observed in the first experiment was seen (Supplementary Fig. 3a). In contrast to the first time-lapse experiment, two of four embryos with normal cell cycle parameters were characterized by multiple nuclei in individual blastomeres (Supplementary Fig. 3b). Interestingly, when we segregated normal four-cell embryos based on appearance of multiple nuclei, we observed that normally developing embryos with one nucleus per blastomere had lower levels of 5mC, while still showing similar levels of 5caC as abnormal embryos (Fig. 4g, h). In summary, our results suggest an important role of DNA methylation reprogramming for normal early human development.

## Discussion

Early human embryogenesis is characterized by low rates of success in development, with only 30–50% of human zygotes reaching the blastocyst stage in vitro. The underlying cause of this poor development is not well understood. In this study, we analyzed the dynamic remodeling of DNA methylation in human preimplantation embryos to further understand the role of epigenetic reprogramming in the success or failure of early human embryogenesis.

In human G2-stage zygotes, our co-staining of DNA 5C modifications with well-characterized antibodies showed a loss of 5mC and the generation of 5hmC in paternal pronuclei, with no significant differences in the later oxidation products 5fC and 5caC in the parental pronuclei (Fig. 1). This indicated a conserved, but less pronounced, active DNA demethylation by TET enzymes in human zygotes compared to the mouse (Supplementary Fig. 1). Notably, we defined the maternal pronucleus as the pronucleus with higher levels of 5mC. This definition has been used in previous studies and was also validated by 5mC staining of polyspermic zygotes (Fulka et al. 2004). Nevertheless, both our observations that (1) 5mC and 5hmC levels are not always asymmetrically distributed in maternal and paternal pronuclei in all analyzed zygotes, but instead show a high heterogeneity at this stage, and that (2) there is little or no difference in 5fC and 5caC levels between parental pronuclei, are not influenced by the definition of the maternal pronucleus applied in this study.

In contrast to the high variability in 5mC and 5hmC levels in the zygote, our IF analysis of two-cell embryos revealed a dissimilar staining pattern of 5mC and 5hmC, staining separate hemispheres of the two-cell nucleus, which is likely attributable to the separation of maternal and paternal chromatin (Fig. 2). This indicates that human DNA methylation reprogramming by TET enzymes is not completed at the zygotic stage, and continues to later preimplantation stages. This finding is further corroborated by the observation that there is no significant difference in 5caC levels in the parental pronuclei of human zygotes (Fig. 1d, e), while human four-cell embryos show mosaic 5caC signals (Fig. 4b). A prolonged first wave of DNA methylation reprogramming in early human embryos is in agreement with a previous study showing a major wave of genome-wide DNA demethylation at the two-cell stage in humans using bisulfite sequencing (Guo et al. 2014a, b; Zhu et al. 2018). Interestingly, we observed high heterogeneity in 5mC and 5hmC signal intensities in the two parental pronuclei of human zygotes (Fig. 1b), which might indicate heterogeneous levels of 5mC in the respective sperm or oocyte methylomes, apart from a failure in TET enzyme-mediated oxidation of 5mC in human zygotes. Indeed, it has been reported that human sperm and oocytes can differ significantly in 5mC levels (Cassuto et al. 2016; Lujan et al. 2019), which can be influenced by environmental factors like aging and nutrition (Ge et al. 2015; Wang et al. 2018). However, the influence of environmental factors on sperm and oocyte methylomes and the newly derived embryo is just beginning to be understood. TET enzymes are dependent on metabolites and co-substrates to function (Yang et al. 2014), which are derived from the environment/nutrition. Lack of sufficient co-substrates for TET enzymes can lead to failure in DNA methylation reprogramming in the germline or the zygote and has the potential to result in the manifestation of epimutations, which could be harmful for embryogenesis and subsequent generations and will require further investigation. In this regard, a recent study reported that hypermethylation of polar bodies derived from human oocytes correlates negatively with successful preimplantation development, with high levels of DNA methylation in specific classes of repetitive elements (Yuan et al. 2021). Together, our results reveal that DNA methylation reprogramming is conserved in human zygotes and follows human-specific dynamics. Moreover, the lack of increased 5fC and 5caC in the paternal genome of human zygotes suggests that both 5fC and 5caC are needed for later processes in preimplantation development and not in the zygote.

Moving forward in development, our analysis of DNA 5C modifications in human cleavage-stage embryos revealed a similar dilution of 5mC, 5hmC, and 5caC in human preimplantation development as reported for the mouse, suggesting passive DNA methylation as the main driver of DNA methylation reprogramming in both species (see also Smith et al. 2014). Interestingly, in contrast to the mouse, we detected a further decrease in 5mC and 5hmC signal intensities in the ICM compared to the TE of early- to late-developing human blastocysts (Fig. 3). A previous study that used bisulfite sequencing of human embryos also showed further demethylation after the morula stage (Guo et al. 2014a, b), and lower DNA methylation levels in the ICM have been reported in other mammals (for bovine see Hou et al. 2007). In this context, differences between human and mouse are also found in embryonic stem cells (ESCs) derived from human and mouse blastocysts, where human ESCs are characterized by a more primed state compared to more naïve pluripotent mouse ESCs, and with the two cell types requiring different culture conditions, which regulate distinct signal pathways. This finding suggests a different strategy of epigenetic reprogramming in human and mouse blastocysts entering the peri- and postimplantation stage and is the subject of ongoing investigations in the field.

Remarkably, our comprehensive approach to gain novel insights into the role of DNA methylation reprogramming in early human embryogenesis revealed a correlation of 5mC and 5hmC levels with normal human preimplantation development. Here, we made use of noninvasive time-lapse imaging to identify normally and abnormally developing human embryos at the four-cell stage by analyzing well-defined cell cycle parameters (Chavez et al. 2012; Vera-Rodriguez et al. 2015; Wong et al. 2010). Strikingly, we detected lower levels of 5mC and 5hmC in normal embryos compared to abnormal embryos (Fig. 4e, f). In contrast, we found highly similar levels of 5caC in normal and abnormal four-cell embryos (Fig. 4g, h), suggesting only a minor function of 5caC in early preimplantation embryos. Our findings of lower 5mC and 5hmC signals in normal four-cell embryos are in contrast to a previous study, which found higher levels of both modifications in normally developing later cleavage-stage embryos (from the six-cell stage onwards) (Petrussa et al. 2016). We speculate that the different sampling conditions might contribute to observed differences (thawed supernumerary zygotes (cryopreserved without quality assessment and equivalent success rates as fresh sibling zygotes; Fugger et al. 1988; Miller and Goldberg 1995), which underwent in vitro culture to the four-cell stage and noninvasive time-lapse imaging to obtain well-defined cell cycle parameters in this study versus cleavage-stage embryos (six-cell stage onwards), which did not fulfill stringent criteria for transfer or cryopreservation on days 3 or 5 (Petrussa et al. 2016)).

The lower 5mC and 5hmC levels detected in normal human four-cell embryos suggest a key role of DNA methylation reprogramming in early human embryogenesis. The differences in acquiring a specific DNA methylome in normally versus abnormally developing human embryos at the four-cell stage correlate with the subsequent timing of embryonic genome activation (EGA), where normal human embryos activate their embryonic genome between the four- and eight-cell stage and abnormally developing human four-cell embryos are characterized by an abnormal transcriptome (Wong et al. 2010). In the mouse, the major wave of EGA occurs at the two-cell stage, correlating with faster dynamics of DNA methylation reprogramming in mouse zygotes (Aoki et al. 1997; Hamatani et al. 2006). Moreover, mouse studies with depletion of TET enzymes show higher variability in their transcriptome, with detrimental outcomes in later embryogenesis (Dai et al. 2016; Kang et al. 2015). Thus, although, we cannot rule out that abnormal development impairs normal methylation reprogramming, it is tempting to speculate that DNA methylation reprogramming in mammals is an important prerequisite for successful EGA. This will require further investigation.

In summary, our analysis of the dynamic appearance of DNA modifications in early human embryos indicates a conserved and essential mechanism of DNA methylation reprogramming by TET enzymes. However, there are pronounced species-specific differences and considerable variability in human compared to mouse preimplantation embryos. Further single-base resolution analysis of DNA methylation reprogramming in normal and abnormal human embryos is needed to identify underlying genomic regions.

## Supplementary data

Supplementary information is available at *Histochemistry and Cell Biology* online.

## Supplementary Information

Supplementary information is available at Histochemistry and Cell Biology online. Below is the link to the electronic supplementary material.

Supplementary file1 (PDF 905 KB)

Supplementary file2 (MP4 14873 KB)

Supplementary file3 (MP4 9956 KB)

## Data Availability

The data underlying this article which are not provided in the supplementary material will be shared by the corresponding author on request.
