# Supplementary material for: Reprogramming of DNA methylation is linked to successful human preimplantation development
Source: Histochem Cell Biol. 2021 Jun 27;156(3):197–207. doi: 10.1007/s00418-021-02008-6 (PMC8460514; doi:10.1007/s00418-021-02008-6)
Supplement: Supplementary file 1 — Supplementary file1 (PDF 905 KB) [file 418_2021_2008_MOESM1_ESM.pdf]

Reprogramming of DNA methylation is linked to successful human preimplantation development

Julia Arand[1,2,3], Renee A. Reijo Pera[2,4,5,#] and Mark Wossidlo[1,2,4,5,$]

[1]Center of Anatomy and Cell Biology, Department of Cell and Developmental Biology, Medical University of Vienna, 1090 Vienna, Austria

[2]Department of Genetics, [3]Pediatrics and [4]Obstetrics & Gynecology, Stanford University, Stanford, CA 94305, USA

[5]Institute for Stem Cell Biology and Regenerative Medicine, Stanford University, Stanford, CA 94305, USA

[#]Current address: McLaughlin Research Institute, Great Falls, MT 59405, USA

[$]Correspondence to: mark.wossidlo@meduniwien.ac.at

Supplementary information list:

Supplementary Fig. 1: Comparison of 5mC, 5hmC, 5fC, and 5caC paternal vs maternal antibody signal ratios of human and mouse zygotes.

Supplementary Fig. 2: 5mC-, 5hmC-, and Nanog-IF in human blastocysts.

Supplementary Fig. 3: 5mC- and 5hmC-IF of time-lapse imaged 4-cell embryos.

Supplementary Table S1: 5mC, 5hmC, 5fC, and 5caC antibody signal ratios of parental pronuclei in human G2-phase zygotes.

Supplementary Table S2: 5mC and 5hmC antibody signal intensities of time-lapse imaged human 4-cell embryos.

Supplementary Table S3: 5mC and 5caC antibody signal intensities of time-lapse imaged human 4-cell embryos.

Supplementary Movie S1: Time-lapse movie (32 fps) of human embryos imaged every 5 min used to determine normal and abnormal cell cycle parameters. Embryos were subsequently analyzed for 5mC and 5hmC by IF.

Supplementary Movie S2: Time-lapse movie (32 fps) of human embryos imaged every 5 min used to determine normal and abnormal cell cycle parameters. Embryos were subsequently analyzed for 5mC and 5caC by IF.

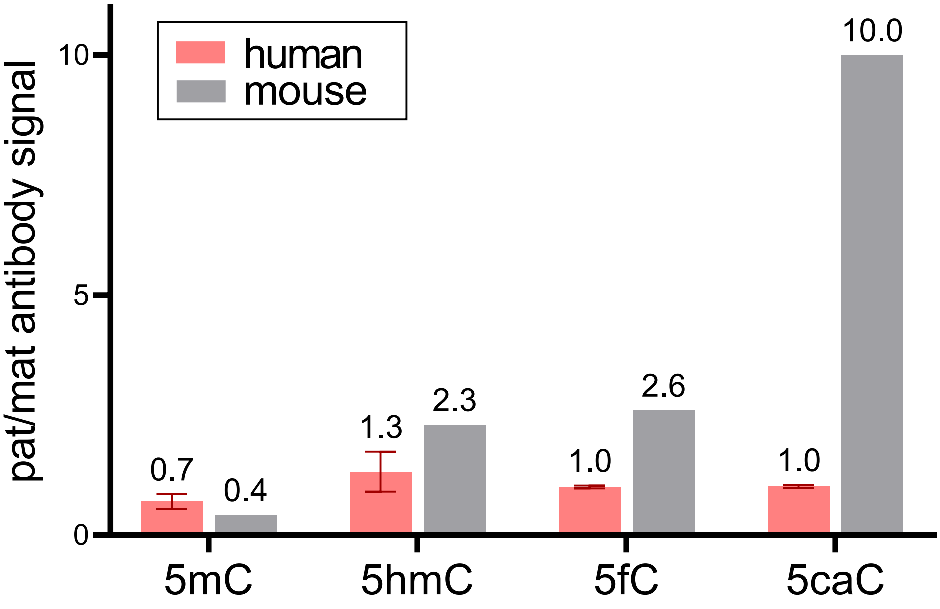

Supplementary Fig. 1: Comparison of 5mC, 5hmC, 5fC, and 5caC paternal vs maternal antibody signal ratios of human and mouse zygotes.

Average signal intensity ratios for mouse zygotes were derived from Wossidlo *et al.* 2011 (5mC and 5hmC) and Inoue *et al.* 2011 (5fC and 5caC).

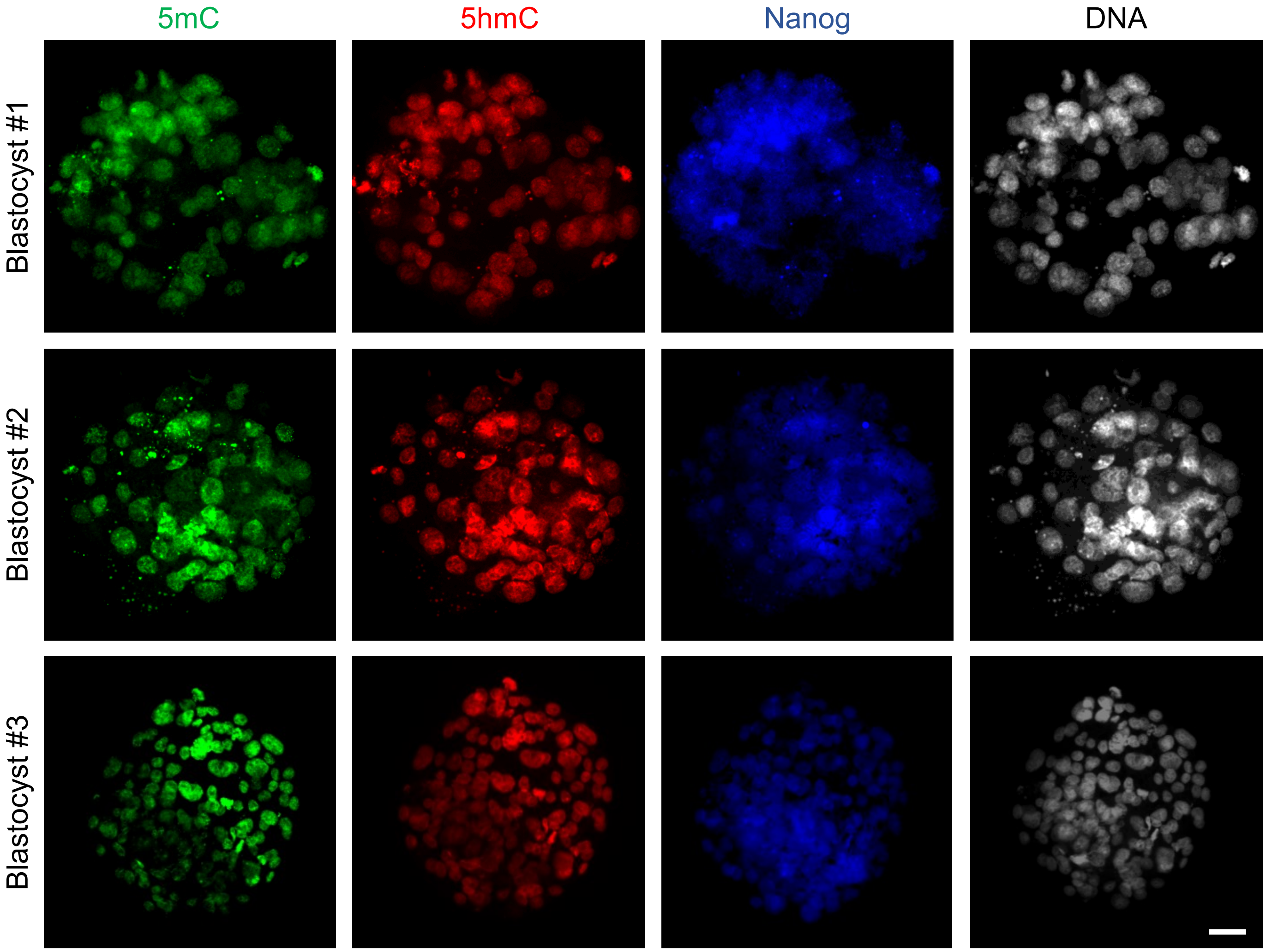

Supplementary Fig. 2: 5mC-, 5hmC-, and Nanog-IF in human blastocysts.

Representative images of human blastocysts co-stained with antibodies against 5mC, 5hmC and Nanog. Stronger 5mC- and 5hmC-signals were observed in the ICM (cells with stronger Nanog signal) in early developing blastocysts #1 and #2, whereas lower 5mC- and 5hmC-signals were detected in the ICM in the late developing blastocyst #3. Note that Nanog staining is of lower quality in these samples, since the blastocysts underwent denaturing conditions for staining with 5mC- and 5hmC-antibodies. Scale bar = 20µm.

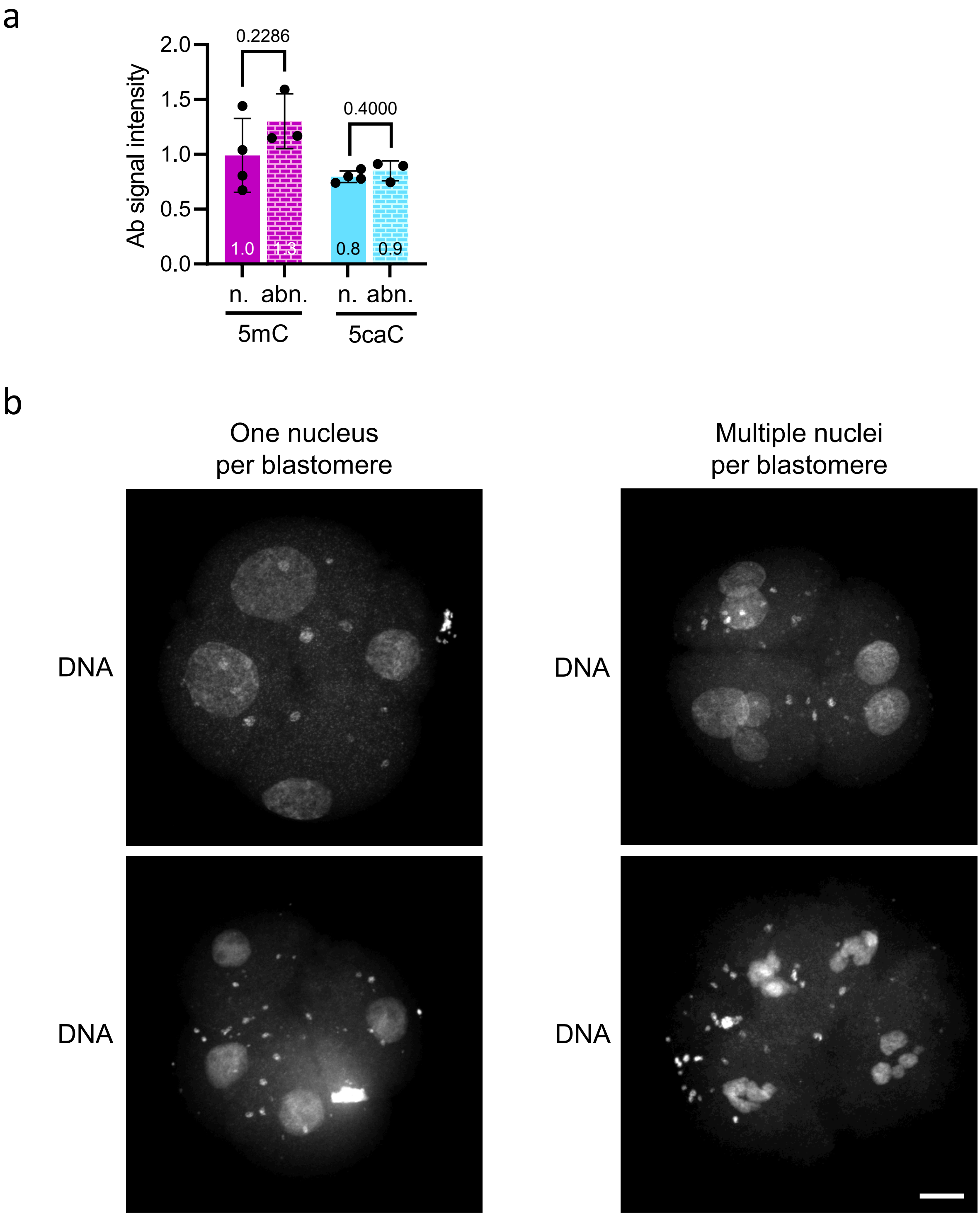

Supplementary Fig. 3: 5mC- and 5hmC-IF of time-lapse imaged 4-cell embryos.

a: Average 5mC- and 5hmC-signal intensities of 4-cell embryos with normal (n.) and abnormal (abn.) cell cycle parameters normalized to DNA signals. Each dot represents a single 4-cell embryo. P-values were calculated using Mann-Whitney test.

b: DNA staining of the four 4-cell embryos with normal cell cycle parameters from panel **a** subdivided into 4-cell embryos with one nucleus or multiple nuclei per blastomere. Scale bar = 20µm.

Supplementary Table S1: 5mC, 5hmC, 5fC, and 5caC antibody signal ratios of parental pronuclei in human G2-phase zygotes.

Co-staining	Embryo	pat/mat 5mC	pat/mat 5hmC	pat/mat 5fC	pat/mat 5caC
5mC & 5hmC	1	0.838	1.283		
	2	0.761	1.403		
	3	0.589	1.332		
	4	0.506	1.012		
	5	0.838	0.939		
	6	0.631	1.130		
	7	0.336	2.187		
5mC & 5fC	8	0.861		1.048	
	9	0.629		1.018	
	10	0.674		0.990	
	11	0.695		0.962	
	12	0.728		1.010	
5mC & 5caC	13	0.553			1.034
	14	0.754			1.017
	15	0.989			1.018
	16	0.650			0.983
	17	0.780			1.007
	18	0.688			1.035
	19	0.559			0.978
	20	0.946			1.077

Supplementary Table S2: 5mC and 5hmC antibody signal intensities of time-lapse imaged human 4-cell embryos.

Embryo	Characteristics	5mC signal	5caC signal
A1	abnormal	1.172	1.407
A2	arrested: 2 blastomeres	na	na
A3	abnormal: 3 blastomeres	na	na
A4	abnormal	0.869	0.786
B1	arrested: zygote	na	na
B2	abnormal: 3 blastomeres	na	na
B3	arrested: zygote	na	na
B4	normal	0.449	0.373
C1	arrested: zygote	na	na
C2	abnormal	1.2	1.001
C3	na (out of focus)	na	na
D1	abnormal	1.041	1.184
D2	abnormal	1.09	1.155
D3	abnormal	1.077	1.37
E1	normal	0.403	0.546
E2	abnormal	1.117	1.486
E3	abnormal	0.858	1.808

Supplementary Table S3: 5mC and 5caC antibody signal intensities of time-lapse imaged human 4-cell embryos.

Embryo	Characteristics	5mC signal	5caC signal
A1	abnormal	1.591	0.895
A2	normal: multiple nuclei	1.041	0.798
B1	abnormal: 5 blastomeres	na	na
B2	normal	0.672	0.867
C1	abnormal	1.168	0.911
C2	abnormal: 3 blastomeres	na	na
D1	arrested: 2 blastomeres	na	na
D2	normal	0.805	0.741
E1	abnormal	1.149	0.746
E2	normal: multiple nuclei	1.44	0.776

Supplementary References

Wossidlo M, Nakamura T, Lepikhov K, Marques CJ, Zakhartchenko V, Boiani M, Arand J, Nakano T, Reik W and Walter J (2011) 5-Hydroxymethylcytosine in the mammalian zygote is linked with epigenetic reprogramming. Nat Commun 2:241. http://doi.org/10.1038/ncomms1240

Inoue A, Shen L, Dai Q, He C and Zhang Y (2011) Generation and replication-dependent dilution of 5fC and 5caC during mouse preimplantation development. Cell Res 21:1670-6. http://doi.org/10.1038/cr.2011.189
